# Intra-Domain Residue Coevolution in Transcription Factors Contributes to DNA Binding Specificity

**DOI:** 10.1128/spectrum.03651-22

**Published:** 2023-03-21

**Authors:** Yizhao Luan, Zehua Tang, Yao He, Zhi Xie

**Affiliations:** a State Key Laboratory of Ophthalmology, Guangdong Provincial Key Laboratory of Ophthalmology and Visual Science, Zhongshan Ophthalmic Center, Sun Yat-sen University, Guangzhou, China; University of Manitoba

**Keywords:** coevolution, DNA binding specificity, DNA-binding domain, transcription factor

## Abstract

Understanding the basis of the DNA-binding specificity of transcription factors (TFs) has been of long-standing interest. Despite extensive efforts to map millions of putative TF binding sequences, identifying the critical determinants for DNA binding specificity remains a major challenge. The coevolution of residues in proteins occurs due to a shared evolutionary history. However, it is unclear how coevolving residues in TFs contribute to DNA binding specificity. Here, we systematically collected publicly available data sets from multiple large-scale high-throughput TF–DNA interaction screening experiments for the major TF families with large numbers of TF members. These families included the Homeobox, HLH, bZIP_1, Ets, HMG_box, ZF-C4, and Zn_clus TFs. We detected TF subclass-determining sites (TSDSs) and showed that the TSDSs were more likely to coevolve with other TSDSs than with non-TSDSs, particularly for the Homeobox, HLH, Ets, bZIP_1, and HMG_box TF families. By *in silico* modeling, we showed that mutation of the highly coevolving residues could significantly reduce the stability of the TF–DNA complex. The distant residues from the DNA interface also contributed to TF–DNA binding activity. Overall, our study gave evidence that coevolved residues relate to transcriptional regulation and provided insights into the potential application of engineered DNA-binding domains and proteins.

**IMPORTANCE** While unraveling DNA-binding specificity of TFs is the key to understanding the basis and molecular mechanism of gene expression regulation, identifying the critical determinants that contribute to DNA binding specificity remains a major challenge. In this study, we provided evidence showing that coevolving residues in TF domains contributed to DNA binding specificity. We demonstrated that the TSDSs were more likely to coevolve with other TSDSs than with non-TSDSs. Mutation of the coevolving residue pairs (CRPs) could significantly reduce the stability of THE TF–DNA complex, and even the distant residues from the DNA interface contribute to TF–DNA binding activity. Collectively, our study expands our knowledge of the interactions among coevolved residues in TFs, tertiary contacting, and functional importance in refined transcriptional regulation. Understanding the impact of coevolving residues in TFs will help understand the details of transcription of gene regulation and advance the application of engineered DNA-binding domains and protein.

## INTRODUCTION

Transcription factors (TFs) regulate target gene expression by recognizing particular DNA sequences and cooperating with other factors, of which functional effects and consequences are often cell-type specific (1, 2). The ability to bind only to specific DNA sequences is often thought to be an indicator of the ability to regulate transcription (1). Despite extensive efforts to map millions of putative TF binding sequences with various high-throughput sequencing methods, identifying the critical determinants contributing to DNA binding specificity remains a major challenge.

The DNA readout of TFs can be affected by many factors. Studies on many TF–DNA structures have demonstrated that TF binding activities can be guided by physical interactions between TF residues and DNA bases (3). For example, the ZBTB member ZBTB24 protein interacts with DNA exclusively in the major groove of one 13-bp consensus motif by forming direct hydrogen bonds, and mutation of residues in the DNA binding domain (DBD) would weaken or even cause loss of its DNA binding ability (4). TFs can also recognize sequence-dependent DNA structures, such as DNA bending (5). For instance, yeast bHLH TFs Cbf1 and Tye7 bind DNA targets with a differential preference for the genomic regions flanking E-box sites according to the DNA shape of the binding sites (6). Another example is the Homeodomain TF Hox-Exd-Hth trimer, which prefers DNA sequences with a complex DNA shape that includes optimally spaced minor groove width minima (7). Moreover, DNA modifications, such as the addition of a methyl group to a cytosine base, can locally modify the structural features of DNA in multiple ways, thereby modulating the interactions with TFs (8, 9). In addition to these DNA-level features, TF binding activity can be influenced by a range of TF-level and chromatin-level features. TF binding specificity can be modulated by intra- and intermolecular TF interactions (10, 11). With regard to chromatin-level features, nucleosome interaction and chromatin accessibility have been illustrated to have the ability to define regulatory elements of TF interaction with DNA (12, 13).

In the last decade, sequence-based high-throughput (HT) technologies to measure protein DNA-binding specificities have revolutionized our ability to measure TF–DNA specificity. Microarray-based assays such as protein-binding microarray (PBM) (14, 15), and sequencing assays such as the bacterial one-hybrid (B1H) system (16), HT systematic evolution of ligands by exponential enrichment (HT-SELEX) (17), and SELEX-seq (18) enable large-scale screening of the DNA binding preferences of TFs. These studies have generally shown that similar TF domains tended to have similar DNA-binding sites and that different TF members had various core binding sites or flanking sequences (15, 19 to 21). Nevertheless, many TFs were found to bind multiple motifs, which makes understanding the binding specificity determinants more challenging.

The coevolution of residuals in TFs may help in understanding the factors contributing to DNA binding specificity according to the clues such as TFs being able to change their motifs, binding partners, and expression patterns during evolution (1). The coevolution of residues is a phenomenon in which residues at one site change depending on the residues of another site (22). Simultaneous changes in residues have been proven helpful in analyzing protein constraints to maintain structural and functional integrity to acquire specific functional necessities (23); understanding protein–protein interaction networks (24, 25); predicting alternative structural conformations and flexibility (26 to 29); and discovering functional residues that play essential roles in the catalytic activity and binding affinity of a protein (30, 31). Previous studies have suggested that the integration of coevolving relationships between TF residues and DNA-binding sites can improve the prediction of substrate specificity (32 to 34).

Despite these studies, whether and to what extent coevolving residues in TF domains contribute to DNA binding specificity is unclear. In the present study, we evaluated the effects of coevolution between residues in the DBDs on TF binding specificity. We systematically collected TF–DNA interactions from HT data sets for nine TF families, including the Homeobox, HLH, Ets, HMG_box, forkhead, bZIP_1, Zn_clus, zf-C4, and zf-C2H2 families. We defined TF subclass-determining sites (TSDSs) for each TF family and showed that the TSDSs coevolved more frequently with other TSDSs than with non-TSDSs. Interestingly, we found some residues coevolving with TSDSs but spatially distant from the DNA interface, which can impact the stability of the TF–DNA complex upon mutation. Collectively, the findings of our study showed that the evolution of residues in TFs played an important role in contributing to the DNA binding specificity of TFs.

## RESULTS

### Characterization of TF binding specificity.

**FIG 1:**
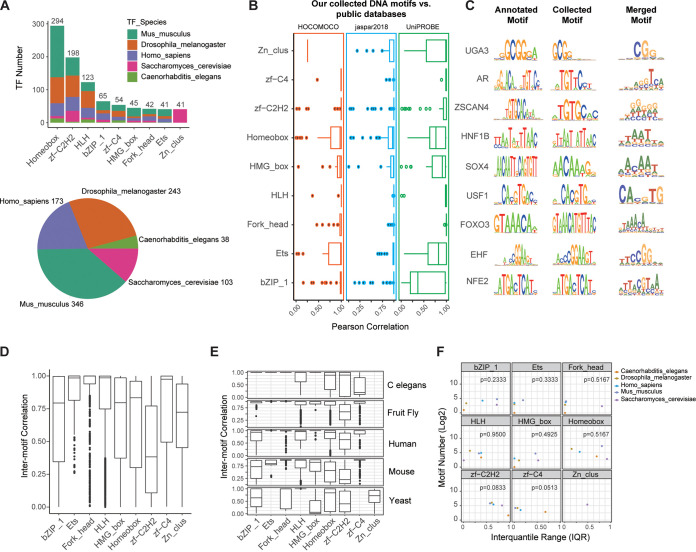
Characterization of TF binding specificity. (A) Overview of TF families. Upper panel: stacked bar plots showing TF numbers for each TF family, where relative percentages of TF in different species are shown with different colors. Lower panel: pie chart showing total numbers of TF in different species. (B) Boxplot for each TF family (left panel) showing the similarity of overlapped DNA motifs between our collected data set and three public databases (HOCOMOCO, JASPAR, and UniPROBE). Sequence logos of representative DNA motifs in public database (middle panel) and our collected data set (right panel) are shown. Information content of each position was used in sequence logos. (C) Sequence logos of aligned DNA motifs in our collected data set for each TF family. Information content of each position was used. (D) Boxplots showing the similarity of DNA motifs between TFs for each TF family. (E) Boxplots showing the similarity of DNA motifs between TFs in each of five species for each TF family. (F) Scatterplot for each TF family showing no correlation between TF numbers and DNA motif diversity in five species. Spearman correlation analysis and tests were performed.

To comprehensively analyze the DNA binding specificity of TFs, we collected publicly available data sets from HT TF–DNA experiments, including PBM, B1H, and SELEX technology (Materials and Methods; Table S1 in the supplemental material). By a unified data processing strategy, we obtained high-quality DNA motifs for 1,179 TFs from mice, humans, fruit flies, yeast, and C. elegans. We discarded the TF families with <30 TF members and finally included DNA binding motifs of 903 TFs in our analyses. These TFs came from nine major TF families, with Homeobox, zf-C2H2, and HLH being the three largest families (Fig. 1A). All 9 TF families were among the top 10 major TF families in animal genomes according to the AnimalTFDB database (35). Except for Zn_clus, all the other families contained TFs from multiple species. Globally, approximately 85% of the TFs were from mice, humans, and fruit flies (Fig. 1A). We compared these collected DNA motifs to several known large-scale databases of TF binding profiles, including JASPAR (36), UniPROBE (37), and HOCOMOCO (38). On average, approximately 68.8% of TFs for each family were found in these databases, with high similarity to the annotated TFs with an averaged Pearson correlation of >0.84 (Fig. 1B). For some TFs, we found nearly identical core consensus sites, such as *NFE2*, *FOXO3*, and *USF1*, in our collected data set compared to those in the known databases (Fig. 1B). These results suggested the reliability of our collected data sets.

The aligning DNA motifs for each TF family converged to a consensus motif (Fig. 1C). While the degenerated DNA motifs showed distinctive binding sites among TF families, the merged DNA motifs for most families showed a weakened consensus at the core or flanking sites, suggesting heterogeneity of DNA-binding sites in the same TF family. We next calculated intermotif similarity scores for all pairs of DNA motifs for each TF family to quantify this heterogeneity. For the TFs from the bZIP_1, HMG_box, Homeobox, zf-C2H2, zf-C4, and Zn_clus families, DNA motif similarity among TFs varied over a wide range (Fig. 1D), reflecting high variability in TF binding sites even within the same family, consistent with the motif alignment results. In particular, the DNA motif similarity between zf-C2H2 TFs was significantly lower than that among the other TF families. Because the TF–DNA interactions we included were from multiple species, the variations in binding preference in a TF family could have been caused by differences among species. We next examined the similarity between the DNA motifs of each TF family for each individual species. A similar pattern was observed (Fig. 1E). In addition, we explored whether the variation between DNA motifs was affected by the number of motifs included by calculating Spearman correlation scores between the interquantile range (IQR) of between-motif similarity and motif numbers for each TF family in each species. For all TF families, no significant associations between higher IQR and motif numbers were observed, which suggested that the higher variation in the similarity between DNA motifs was unlikely to have been caused by the sample size (Fig. 1F; Spearman correlation test, all *P > *0.05). Together, these results demonstrated that DNA binding preference was divergent between different TF families and that even TFs within the same family exhibited heterogeneity in DNA binding specificity to various degrees.

### Identification of TF subclass determining sites (TSDSs).

We next grouped the TFs into different subclasses according to the DNA motif similarity and defined the TSDSs for each family (see Materials and Methods). We noticed that the zf-C2H2 family contained more than 20 subclasses, which made the size of each subclass too small and was consistent with the fact that zf-C2H2 TFs usually contain multiple DBD copies as an array, which allows the TFs to recognize new binding sites (1, 39). Meanwhile, the forkhead TFs showed high similarity between DNA motifs, resulting in only one subclass. We discarded these two TF families before conducting further analysis.

**FIG 2:**
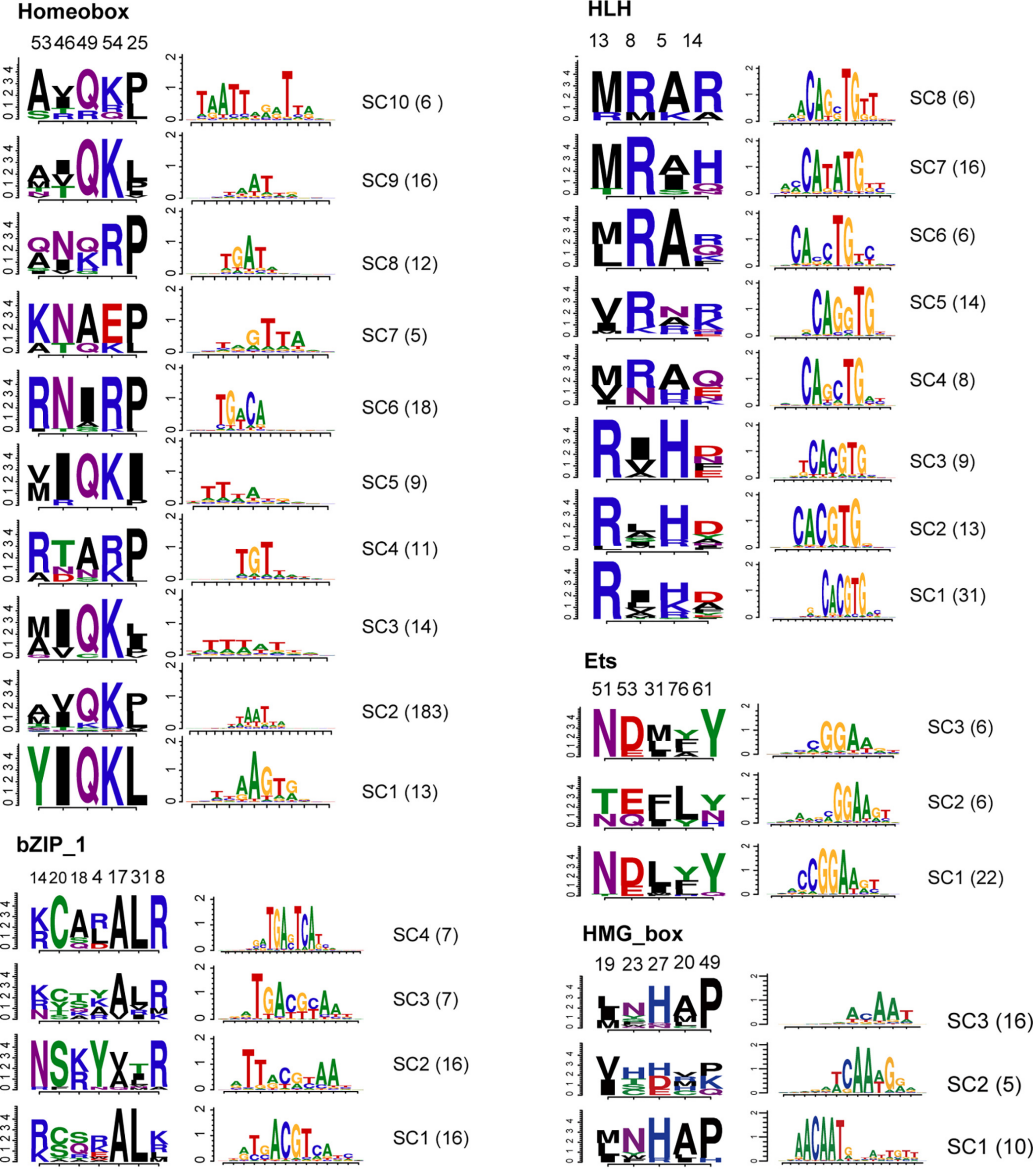
Identification of TF subclass determining sites (TSDSs). Sequence logos of TSDSs (left panel) and corresponding merged DNA motifs (right panel) for each TF subclass from five TF families: Homeobox, HLH, bZIP-1, Ets, and HMG_box. The number of members of each subgroup is shown in the parenthesis. Information content of each position was used in sequence logos.

Our analyses reproduced many known TSDSs or TF subclasses (Fig. 2, Fig. S3). For instance, we found that 62% of Homeobox TFs bind the typical DNA motif “TAAT” (40). Of the five TSDSs, four (53, 46, 49, and 54) were located in the recognition helix. Moreover, the residue 49 has been demonstrated to be crucial for specific DNA binding with mutation assays (41). For the HLH family, we found a significant DNA consensus sequence, “CANNTG,”’ known as the “E-box,” which is recognized by almost all TFs (42). Interestingly, four positions (5, 8, 13, and 14) on the HLH domain were related to different forms of the E-box, among which Arg13R was enriched in TFs binding the CACGTG motif, consistent with the half-site-based analysis (43). For Ets TFs, we found five DBD positions (31, 51, 53, 61, and 76) related to DNA binding specificity, where positions 51 and 53 were on helix 3 of the domain and position 76 was on strand 4 (44). For HMG_box TFs, five positions (19, 20, 23, 27, and 49) were informative for subclasses, where positions 19, 20, and 23 were on alpha helix 1, and position 27 was at the N terminus of alpha helix 2, known as typical structures of the HMG-box domain (45). For bZIP_1 TFs, seven DBD positions (4, 8, 14, 17, 18, 20, and 31) were correlated with TF subclusters, where positions 17 and 20 were known to be signatures for DNA recognition (46). Together, these results indicated that our analysis revealed a reliable relationship between TF subclasses and their specific DNA binding activity.

We noticed that defining TF subclasses usually required more than one TSDS. For example, we showed that the well-studied DBD position 49 in Homeobox TFs was not present in all subclasses, while the residues 53R and 49Q were found in different TF subclasses (Fig. 2). Taking HLH TFs as another example, 13R and 8R also appeared to be mutually exclusive. Combining 8R and the amino acids at the other positions accounted for the divergence of noncanonical E-boxes, such as CATGTG and CAGGTG. These results suggested that the TSDS combination can improve the accuracy in predicting DNA binding specificity, and TSDS positions tended to covary in terms of amino acid composition.

### Coevolving residue pairs (CRPs) and TSDSs.

Correlated mutations or covariation between residues were thought to be suggestive of coevolution (47). We next explored whether and to what degree these TSDSs coevolved in correspondence with DNA binding specificity. Researchers have designed many statistics and computational methods to measure coevolution events observed in homologous sequences or in functionally relevant sequences. This complicates the validation of any measure of indirect evidence. In this study, we employed two strategies to reduce undesirable effects. First, we compiled an independent data set containing a total of 123,976 TF-domain sequences from 432 species from the Pfam database, which were subjected to a realignment with the same seed sequence for each TF family. Second, because a variety of methods have been developed to quantify the coevolution of protein residues and conflicts may exist between different methods, we predicted the coevolving residue pairs with high likelihood by combining multiple methods, including MI, MIp, SCA, and OMES, to detect reliable CRPs (see Materials and Methods). The CRP candidates were defined with the top 10% residue pairs for each method. In general, MI, Mip, and OMES yielded moderately to highly consistent results with Jaccard similarity coefficients ranging between 0.43 and 0.83, while the SCA measurement weakly correlated with all the other methods (Fig. S4), which was consistent with a previous benchmarking study (48). We also compared the MIp values used in our study to those from the MISTIC webserver, another popular mutual information server to infer coevolution. We found a high correlation between these two platforms, with a median Pearson correlation coefficient of 0.7 (Fig. S5).

**FIG 3:**
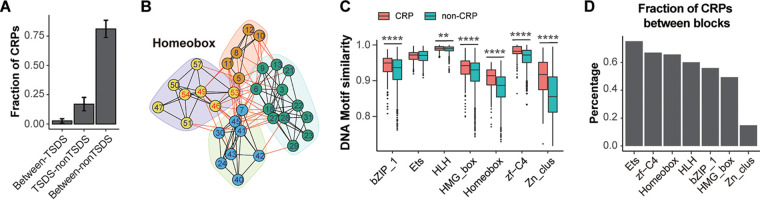
CRPs and TSDSs. (A) Bar plots showing the fraction of CRPs between TSDSs, between TSDSs and non-TSDSs, and between non-TSDSs across all TF families. (B) Representative network-based partition of coevolving residues in Homeobox. Nodes and numbers refer to residue index in the TF domain; edges refer to coevolving relationship. Numbers in red indicate TSDSs. Nodes in different clusters are shown in different colored background. (C) Boxplots showing comparison of DNA motif similarity of TFs grouped by CRPs and non-CRPs. *t* tests were conducted in statistical testing. **, *P* < 0.01; ****, *P* < 0.0001. (D) Bar plots showing the fraction of out-of-block coevolution for each TF family.

Final CRPs were defined with the candidates identified by at least two methods for each TF family (Table S2). We revealed CRPs between TSDSs and/or non-TSDSs. As non-TSDSs accounted for most of the residues in the TF domains, approximately 81% of the CRPs were among non-TSDSs on average, while approximately 2.7% of the CRPs were among TSDSs in Homeobox, HLH, bZIP_1, Ets, and HMG_box TFs (Fig. 3A). Interestingly, in these five TF families, we found that TSDSs tended to more frequently coevolve with TSDSs, as revealed by network-based community analysis showing that the TSDSs were usually clustered (Fig. 3B; Fig. S5). A similar clustering relationship was also found using CRPs identified with the DCA method. To investigate the influence on the DNA binding specificity of CRPs, we compared the DNA motif similarity between TFs grouped by CRPs or by non-CRPs (see Materials and Methods). Interestingly, we found that, except for Ets TFs, the TFs grouped by CRPs had a higher degree of DNA motif similarity than those grouped by non-CRPs in the six TF families out of the seven we tested (Fig. 3C), suggesting that the CRPs were related to similar DNA binding activities.

We next examined whether the CRPs were adjacent in the sequence of amino acids, known as blocks, that were considered to be important in protein evolution (49). We found that more than half of the CRPs were between residues more than five positions apart from each other in the alignment of six TF families, except in the Zn_clus TFs (Fig. S6). By defining the residue blocks (Table S3), we further found that as many as 75% of CRPs were between different blocks in bZIP_1, Ets, HLH, HMG_box, Homeobox, and zf-C4 TFs, while the out-of-block coevolving pairs only accounted for ~15% of CRPs in Zn_clus TFs (Fig. 3D), suggesting that the CRPs were not always continuous in the DBD.

### CRPs and TSDSs in the TF–DNA complex.

**FIG 4:**
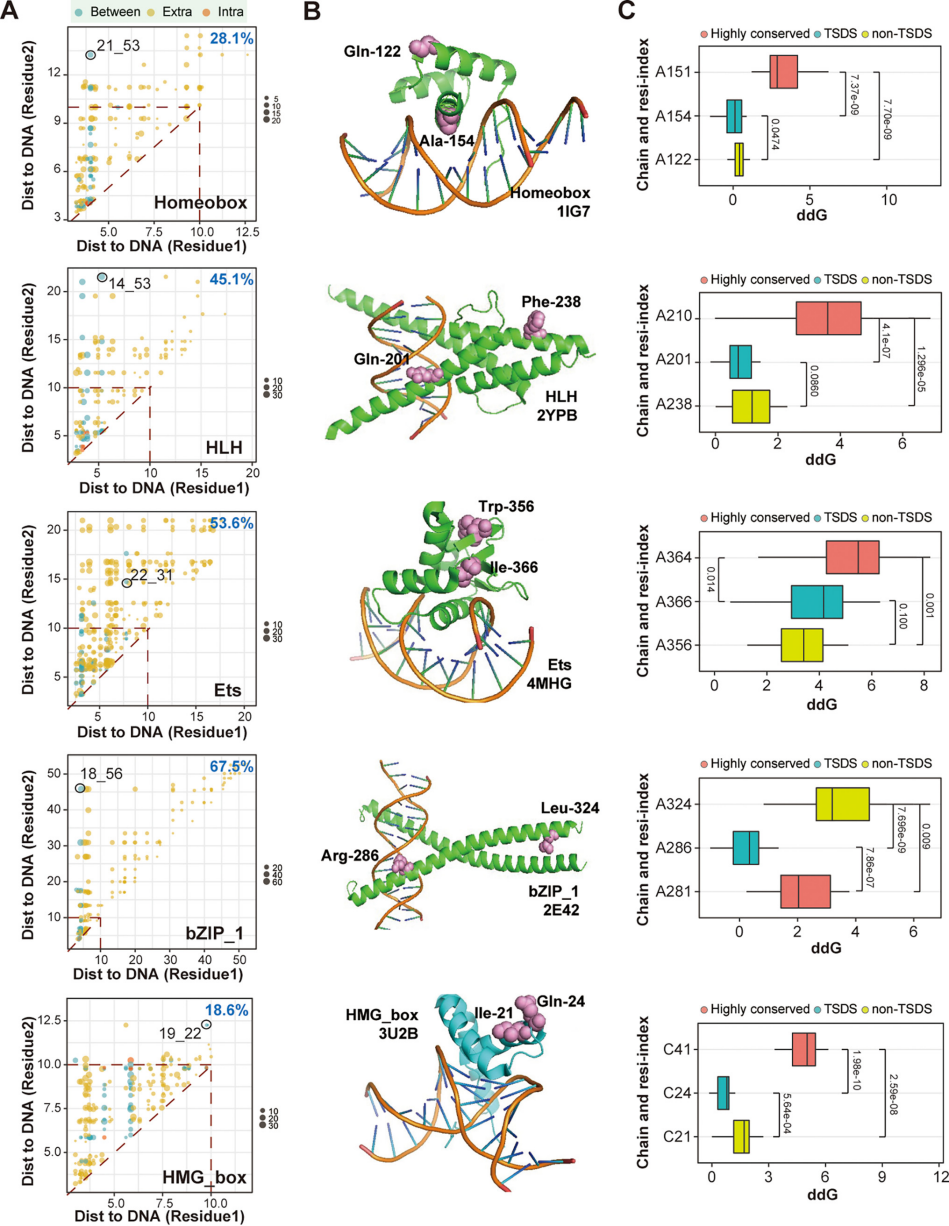
CRPs and TSDSs in TF–DNA complex. (A) Scatterplots showing comparison of distance to DNA interface of each residue in CRPs in Homeobox, HLH, Ets, bZIP_1, and HMG_box TFs. The CRPs from different groups of residue pairs between TSDSs (intra), between TSDSs and non-TSDSs (between), and between non-TSDSs (extra) are in different colors. For each TF family, the percentage of CRPs having at least one residue with a distance of >10A to DNA is calculated. Representative CRPs between TSDSs and non-TSDSs are highlighted in circles and noted with a residue index. (B) Representative PDB structures for Homeobox (1IG7), HLH (2YPB), Ets (4MHG), bZIP_1 (2E42) and HMG_box (3U2B) families. Representative CRPs shown in panel A are highlighted in red spheres and noted with amino acid type and residue ID within one side chain containing TF domain. (C) Boxplots showing the ΔΔG of mutants of indicated residues in selected CRPs by comparing with wild type of selected PDB structures in Homeobox, HLH, Ets, bZIP_1, and HMG_box TFs. Side chain of amino acid and residue IDs are used to indicate the residue mutant. One-tailed Wilcoxon tests were conducted in statistical testing.

In addition to the residue blocks conveying coevolution constraints, TF residues located in the TF–DNA interface are also likely to impact DNA binding (45). Thus, we investigated the structural location of the CRPs and TSDSs in the TF–DNA complex. We collected 178 TF–DNA complexes with a resolution cutoff (4 Å) from the PDB database for seven TF families (Table S4; see Materials and Methods), of which the side chains with the DBD were aligned individually. By mapping TF residues to 3D structures, we estimated the spatial distance of all pairs of DBD residues (see Materials and Methods). While the CRPs were globally located closer in 3D structures than the non-CRPs (*t* test, *P < *10^−8^ for all TF families; Fig. S7), approximately 31% of CRPs on average across seven families had a spatial distance of >10 Å, which was consistent with previous findings that not all the coevolving residues were close together in the 3D structures (50). Comparing the distance to the DNA interface of each residue in CRP pairs in each TF family, we found that the percentages of CRPs with at least one residue far away from the DNA interface (f-CRPs), with a distance of >10 Å, ranged from 18.6% to 67.5%, with a median of 33.3% (Fig. 4A), suggesting heterogeneity in the contribution of CRPs to DNA binding activity in different TF families. Among these f-CRPs, most were between non-TSDSs, while several were between TSDSs and non-TSDSs in Homeobox, HLH, Ets, bZIP_1, and HMG_box TFs. Of note, the distance to DNA of the TF residue off the interface varied over a wide range, with distances as far as >50 Å in bZIP_1 TFs (Fig. 4A). In addition to 4 Å as a resolution cutoff, we also used TF–DNA complexes with a cutoff of 2.5 Å. We found high correlations of the spatial distance measurements between the data sets using different cutoff values for all the TF families (Pearson correlation tests, all *P < *2.2e-16). These results indicated that our results would not be affected by the choice of resolution cutoff in the TF–DNA complex structure data.

To analyze whether the f-CRPs can impact DNA binding, we estimated the changes in interaction energy (ΔΔ*G*) upon mutation of the residues in TF–DNA structures using FoldX, which is a very popular toolset that provides a fast and quantitative estimation of the importance of the interactions contributing to the stability of proteins and protein complexes (see Materials and Methods). We conducted *in silico* modeling with mutation analyses on selected f-CRPs from representative PDB structures (PDB: 1IG7 for Homeobox; 2YPB for HLH; 4MHG for Ets; 2E42 for bZIP_1; 3U2B for HMG_box) from the five families in which we identified known TSDSs (Fig. 4B). Each CRP was between a TSDS and a non-TSDS (Fig. 4A); thus, we could compare the impact of TSDS and non-TSDS mutations on TF–DNA binding activity. We also conducted mutation analysis of highly conserved amino acid sites and compared them with those of the CRPs. We found that the mutations of highly conserved residues induced significantly higher ΔΔ*G* than the other tested mutations in five out of six TF families, including the Homeobox, HLH, Ets, and HMG_box families (Fig. 4C). Of note, we found that the mutant of non-TSDS residues off the DNA interface induced significantly higher ΔΔ*G* than that of TSDS residues close to the DNA interface in the Homeobox, HLH, bZIP_1, and HMG_box TFs (Wilcoxon test, all *P* < 0.1). In addition, in Ets TFs, the mutation of either TSDS or non-TSDS induced a ΔΔG of >2 kcal/mol, which was generally thought to be enough to completely disrupt the DNA binding capabilities (43). These results together demonstrated the biological effects on DNA binding of coevolving TF residues, even for those spatially distant residues from the DNA interface.

## DISCUSSION

Understanding the basis of the DNA-binding specificity of TFs has been of long-standing interest. In this study, we provided evidence that coevolving residues in TF domains contributed to DNA binding specificity. We demonstrated that the TSDSs were more likely to coevolve with other TSDSs than with non-TSDSs. Mutation of the CRPs could significantly reduce the stability of the TF–DNA complex, and even distant residues from the DNA interface contributed to TF–DNA binding activity. This study expands our knowledge of the interactions between coevolved residues in TFs, tertiary contact, and the functional importance in refined transcriptional regulation. Understanding the impact of coevolving residues in TFs will help in understanding the details of transcription for gene regulation and will advance the application of engineered DNA-binding domains and proteins.

While TF preferences for specific DNA binding motifs have been well studied and are thought to be one primary regulatory mode, recent studies have elucidated additional layers that modulate TF–DNA binding, including TF–TF interactions, TF-cofactor interactions, DNA modifications, DNA shape, genomic context, and even genomic variations (51). Interestingly, our study demonstrates that coevolving residues in TF domains can also be used to guide the fine-tuning of TF–DNA binding, which expands the additional layers beyond the DNA binding motifs. Moreover, we found that specific DNA binding activity is the result of a combination of multifaceted regulations, such as those related to DNA motifs and coevolving TF residues, as revealed by this study. It is worth noting that covarying residue pairs within a protein are not necessarily a result of residue proximity in the 3D structure. Confounding residue correlations can also reflect constraints on residues involved in oligomerization, protein–protein, or protein–substrate interactions or other spatially indirect effects, including entropic effects and competition between sites (52 to 55). For example, this combinatorial effect can also be achieved by combining other factors, such as DNA motifs and DNA shape (56), DNA methylation, and structural context (9). Further studies are required to explore whether the coevolving residues in TFs relate to the interactions between TFs and other molecules (TFs and cofactors) in TF–DNA binding.

The probability and stability of TF binding to particular DNA sequences can be modeled with a function of the free energy (57, 58). By estimating the changes in the Gibbs free energy of binding between TF mutants and DNA sequences, we showed the importance of coevolving residues in the structural integrity and DNA binding specificity of TFs with *in silico* mutation analysis of representative TF–DNA structures from five TF families. Our analysis revealed the residues that are distant from the DNA interface but show considerable impacts on DNA binding compatibility upon mutation. This was consistent with the findings of a recent study showing that many combinations of mutations to poorly conserved TF residues and nucleotides flanking the core binding site alter but preserve physiological binding, by measuring affinities for approximately 210 mutants of a model yeast TF interacting with 9 oligonucleotides (59). These results support the existence of a mechanism by which combinations of *cis* and *trans* mutations could modulate the fine-tuning transcriptional regulation during evolution.

We noticed that our TSDSs did not recover all the known amino acid sites related to DNA binding specificity, such as the flexible N-terminal arm of the homeodomain, which can show base-specific contacts with the minor groove via conserved arginine in this region (60). The reasons could be that our analyses were based on the monomers and excluded highly conserved residues from the domain multiple sequence alignment (MSA) profiles. Further studies are required that integrate complex interactions between TF domains, such as homodimers and heterodimers of TFs.

## MATERIALS AND METHODS

### Data collection and processing.

We collected TF–DNA interaction data sets (Table S1) for several major species, including mouse (Mus musculus), human (Homo sapiens), fruit fly (Drosophila melanogaster), yeast (Saccharomyces cerevisiae), and Caenorhabditis elegans. Data from ChIP-Seq-based experiments were not included because of possible confounding by TF partners. DNA motifs were presented using position weight matrices (PWMs) (61). An annotated collection of public databases for TF binding profiles was used to compare our collected DNA motifs to the known motifs with the MotifDb package (62), where HOCOMOCOv11, JASPAR2018, and UniPROBE were selected. DNA motifs for each TF family were aligned and merged with the “*DNAmotifAlignment”* function from the motifStack package (63). TF domain sequences were defined based on the Pfam database (64). MSA of TF domain sequences was conducted by MUSCLE (v3.8.31), one of the most popular computer programs for creating multiple alignments of protein sequences, with the default parameter list except for the input fasta file (65). In amino acid sequence MSA, the typical domain sequence or seed sequence obtained from the Pfam database was used as the constraint. Sequence logos of the MSAs of amino acid sequences were generated with WebLogo3 (66), in which the information content in bits for each position was visualized and the height of the symbols within the stack indicated the relative frequency of each amino or nucleic acid at that position (Fig. S1, S2).

### Identification of TF subclass determining sites (TSDSs) of TF.

Each TF family was grouped into subclasses by hierarchical clustering analyses based on the pairwise similarity of DNA motifs. The similarity measured by the Pearson correlation coefficient between DNA motifs was estimated with the “*motifDistances”* function from the MotIV package (67), which facilitates and extends the use of STAMP (68) in the R environment and command-line processing workflow for comparing a set of motifs to a given database. The number of TF subclusters was determined according to the elbow method. Clusters with a within-cluster sum of squares (WSS) lower than 10% of the starting cluster without partitioning were used. The subclasses containing <5 TFs were excluded from further analysis. TSDSs were identified using the standalone version of SPEER-SERVER, which was an algorithm showing better performance than most TSDS detection methods (69). The SPEER algorithm predicts TSDS by analyzing quantitative measures of the conservation patterns of protein sites based on their physicochemical properties and the heterogeneity of evolutionary changes between and within the protein subfamilies. In SPEER runs, we assigned equal weights to relative entropy, Euclidean distance, and the evolutionary rate of input MSA. The MSA columns with 100% identity in all TF sequences were excluded because they were unlikely to be related to specificity. TSDSs were defined as residues with a *P* value of <0.1 in SPEER runs.

### Coevolution analyses of residues.

Coevolution analyses of residues were performed using TF domain MSA collected from the Pfam database. Four different algorithms were applied, including mutual information (MI) (70), MIp (71), statistical coupling analysis (SCA) (72), and OMES (73). Specifically, MI measures the reduction of uncertainty in one position by considering the information of the other, thus quantifying between-residue covariation; MIp is an adjusted MI by removal of the background MSA phylogenetic signal; SCA measures statistical interactions between amino acid positions to map energetic interactions; OMES detects differences between observed versus expected frequencies of residue pairs. All these algorithms were performed with the “*Evol*” module of ProDy (74). Coevolution scores from four algorithms were combined following the strategy: score values between residue pairs from each method were rescaled by formula $\left(x_{i}\;-\;x_{\text{min}})/(x_{\text{max}}\;-\;x_{\text{min}}\right)$, where *x*_i_, *x*_min_, and *x*_max_ indicated the score for the *i*-th pair, the minimal score. and the maximal score, respectively; rescaled scores were subjected to quantile normalization; and normalized scores for each residue pair were then averaged.

Coevolution scores were compared between the groups with Wilcoxon tests, and the false discovery rate (FDR) was used to correct the *P* values from multiple testing. A *P* value of 0.05 was taken as the cutoff for statistical significance. The coevolving network for each TF family was constructed by taking the residue positions in the MSA as nodes and coevolving relationships as edges. Network analyses and visualization were conducted with igraph (https://igraph.org/). The fast-greedy modularity optimization algorithm was used to detect community structure (75). To estimate the effects of coevolution on DNA binding specificity, we calculated the similarity scores between the DNA motifs corresponding to TFs containing specific amino acid pairs within the CRPs. The DNA motif similarity scores were then summed using the ratio of specific amino acid pairs as a weight vector.

### TF–DNA complex structure analyses and computational mutation analyses.

TF–DNA structures were first collected from the Pfam database. We downloaded the structures with a resolution of <4 Å and containing both amino acid and DNA chains from the PDB database (http://www.rcsb.org) (76). All amino acid chains in PDB structures were included and aligned to the same reference sequence. The distances between protein residues and/or nucleotides were quantified with the shortest Euclidean distance between atoms in the resolved TF–DNA complex using the Rpdb package, which provides tools to read, write, and visualize PDB files and to perform some structural manipulations (77). TF–DNA base contact and the stability of the TF–DNA complex upon mutation of amino acids or DNA bases were predicted with the protein design tool FoldX version 4 (78). We chose FoldX because the predictive power of this tool has been tested on a very large set of point mutants (1,088 mutants) spanning most of the structural environments found in proteins. The PDB structures were first repaired with the “*RepairPDB”* command. Next, phenotypes of the DNA mutant were predicted with the “*DNAScan”* command, and those of the protein residue mutant were predicted with the “*BuildModel”* command. Foldx simulations were performed for each mutant five times to increase the conformational space explored, and the averages were reported. Visualizations of the TF–DNA complex were conducted with Edu PyMol (the PyMOL Molecular Graphics System, v1.7.4, Schrödinger, LLC).

### Data availability.

All the data set and code files used in this study are available at the Github repository with the link https://github.com/trumanLuan/pdi.
