# Supplementary material for: Intra-Domain Residue Coevolution in Transcription Factors Contributes to DNA Binding Specificity
Source: Microbiol Spectr. 2023 Mar 21;11(2):e03651-22. doi: 10.1128/spectrum.03651-22 (PMC10100741; doi:10.1128/spectrum.03651-22)
Supplement: Supplemental file 1 — Supplemental material. Download spectrum.03651-22-s0001.pdf, PDF file, 1.0 MB [file spectrum.03651-22-s0001.pdf]

Supporting Figures and legends

Figure S1. Sequence Logos of aligned DNA binding domains in our analysis for each TF family.

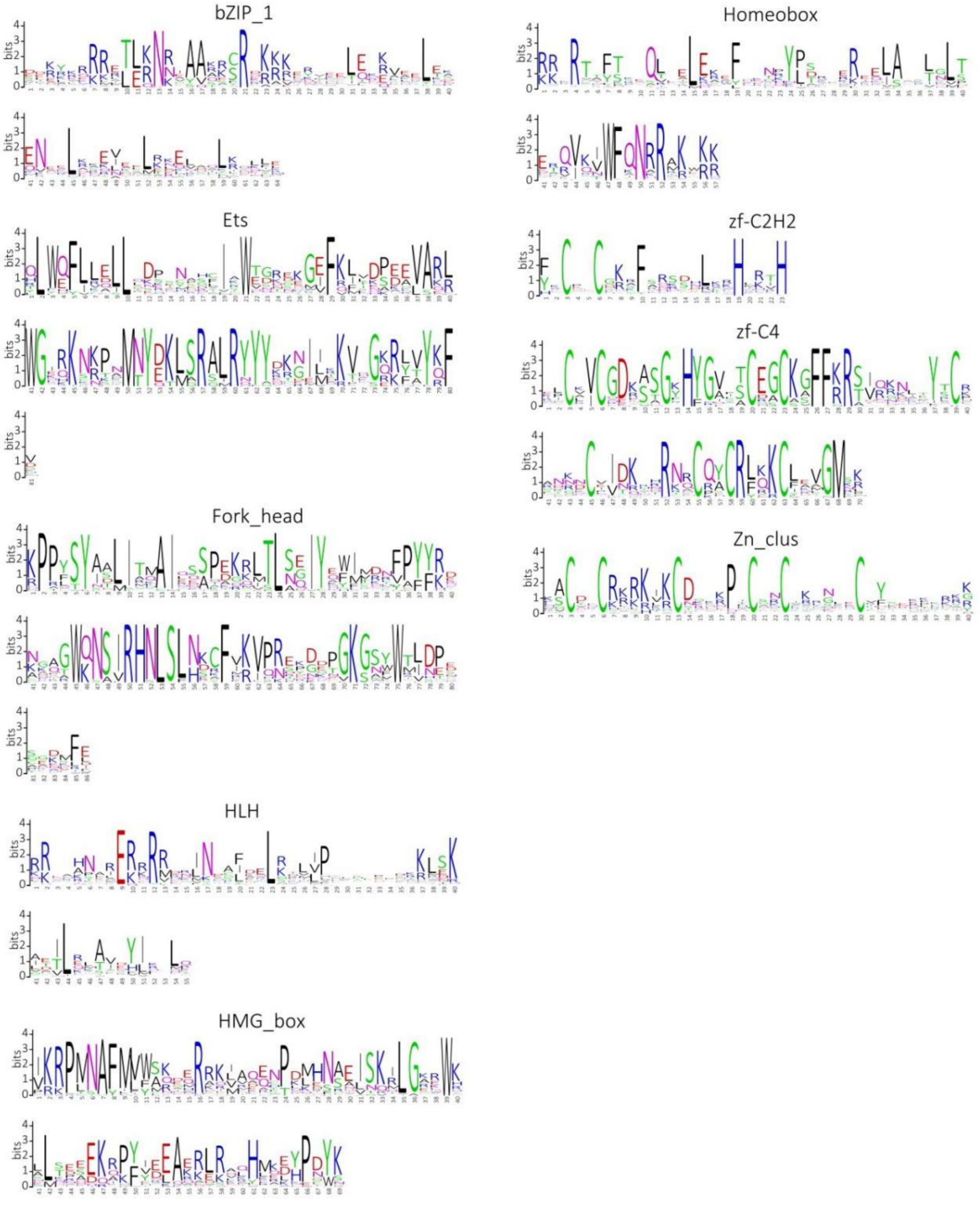

Sequence logos of the MSAs of DNA binding domains of TFs that are included in our collected datasets for each TF family.

Figure S2. Sequence Logos of DNA binding domains for each TF family in Pfam.

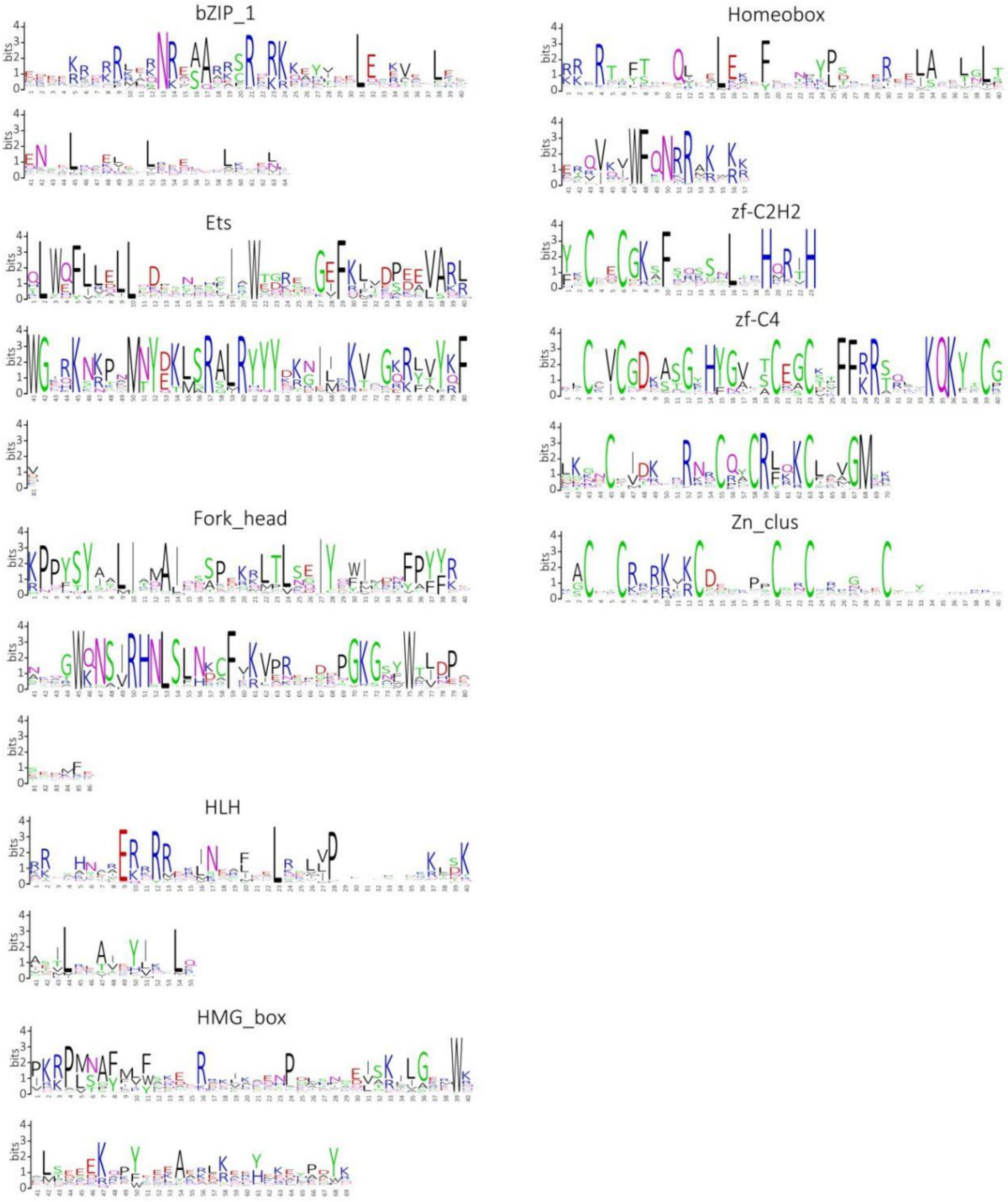

Sequence logos of the MSAs of DNA binding domains of TFs in the Pfam database for each TF family.

Figure S3 TF subgroup determining sites in subfamilies and corresponding DNA motifs.

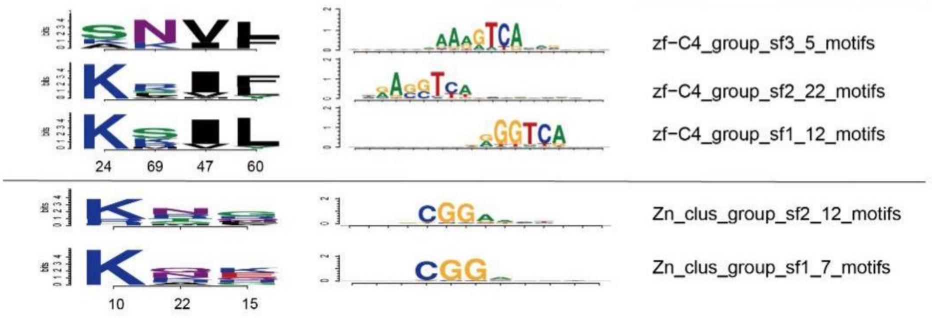

Sequence logos of TSDSs (left panel) and corresponding merged DNA motifs (right panel) for each TF subclass from two TF families: zf-C4 and Zn_clus. The number of members of each subgroup is shown in the parenthesis. Information content of each position was used in sequence logos.

Figure S4 Jaccard-based correlation of four coevolution methods.

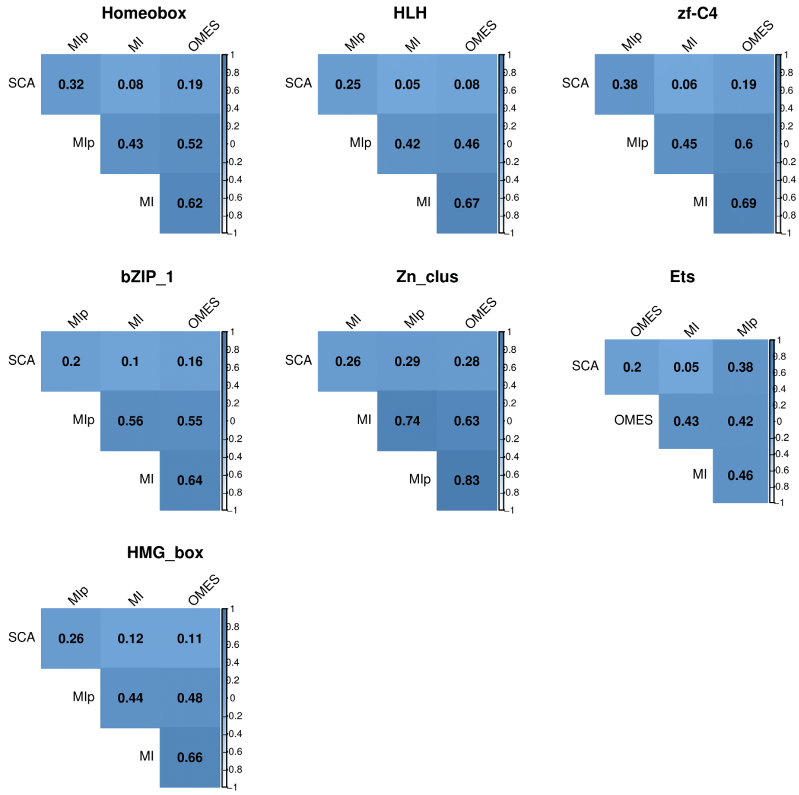

Jaccard-based correlation of four coevolution methods for each TF family.

Figure S5

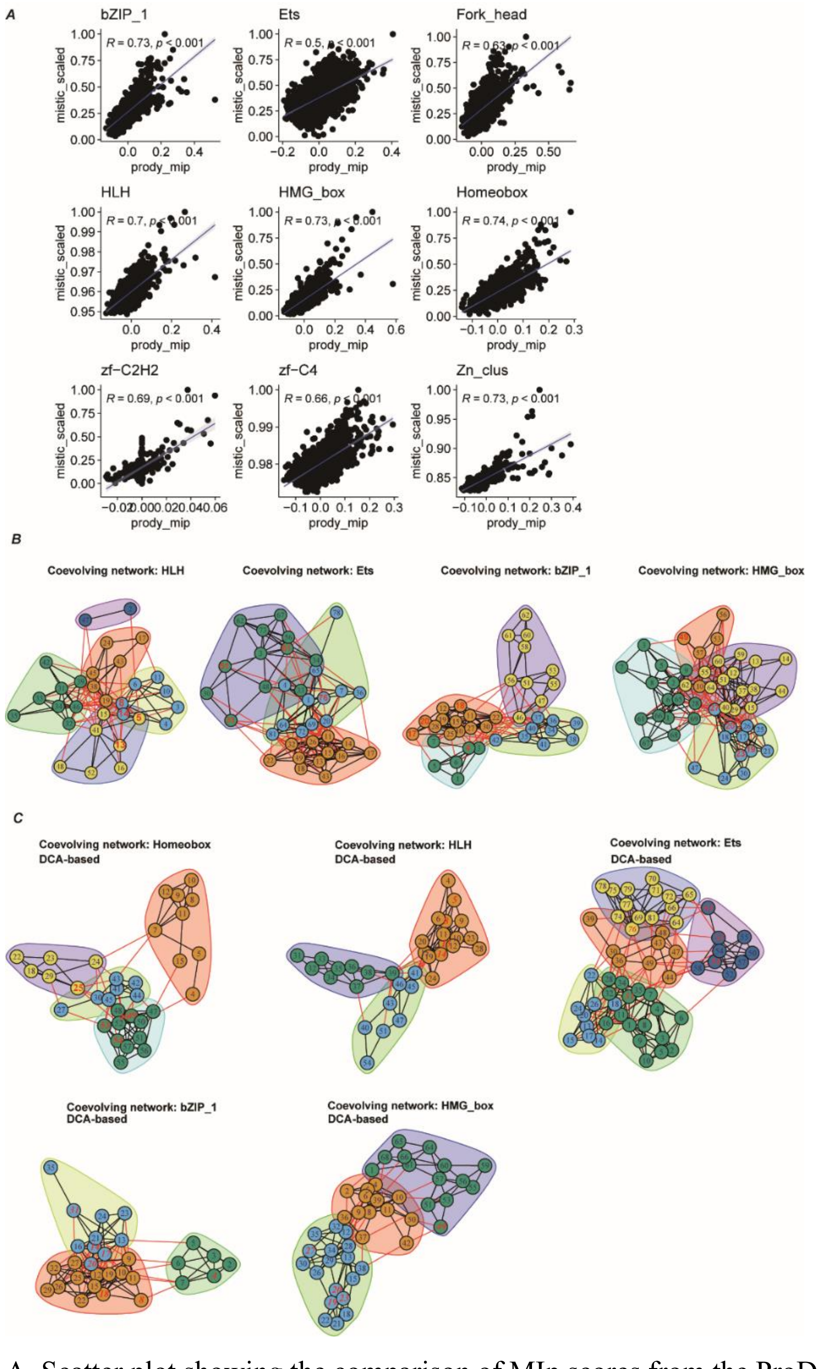

A, Scatter plot showing the comparison of MIp scores from the ProDy platform and MI scores from the MISTIC webserver. Pearson correlation testing was performed.

B, Network communities of coevolving residues for each TF families, related to Figure 3E. The positions in the MSAs for the TSDSs are colored in red.

Figure S6. The coevolving residue distance within the MSA profile.

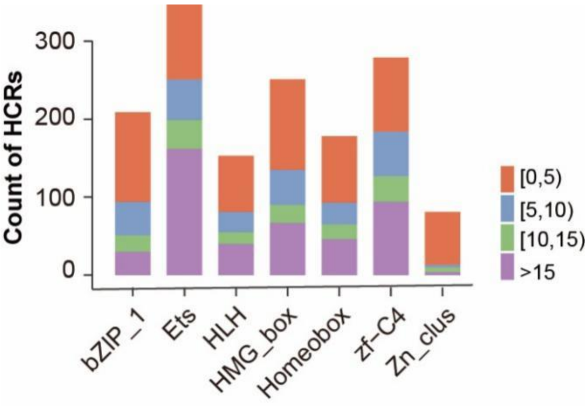

Overview of the distance along the MSA profiles between the CRPs.

Figure S7. Comparing the distance between coevolving residue pairs (CRPs) and non-CRPs.

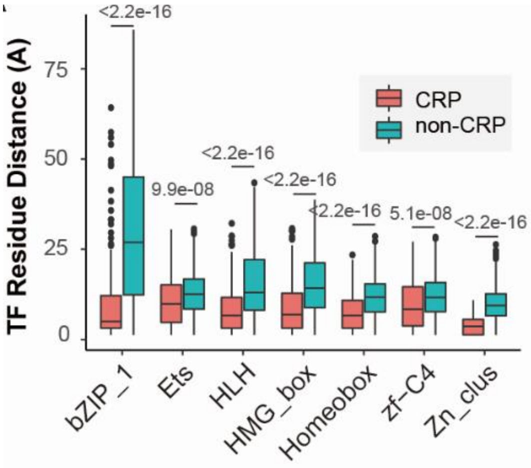

Comparing the spatial distance between coevolving residue pairs (CRPs) and non-CRPs.

Supplementary Tables

Table S1. Overview of included TF-DNA interaction assays

Source	Data type	PMID	# TFs	Description
Berger_2006	PBM	16998473	4	TFs from yeast, worm, mouse and human
Berger_2008	PBM	18585359	157	Homeobox proteins in mouse
Badis_2008	PBM	19111667	110	HLH, bZIP_1, zf, Fork_head, Homeobox in yeast
Scharer_2009	PBM	19147588	1	HMG_box in human
Zhu_2009	PBM	19158363	29	bZIP_1, HLH, zf, fork_head, HMG_box in yeast
Lesch_2009	PBM	19204119	1	Homeobox in C. elegans
Badis_2009	PBM	19443739	101	HLH, bZIP_1, zf, Ets, fork_head, HMG_box in mouse
Grove_2009	PBM	19632181	10	HLH in C. elegans
Jolma_2013	SELEX	23332764	258	HLH, bZIP_1, zf, Ets, fork_head, HMG_box, Homeobox in human
Weirauch MT_2014	PBM	25215497	932	TFs in Arabidopsis, mouse, C. elegans, human and fly
FlyFactorSurvey	B1H	21097781	297	HLH, zf, bZIP_1, Ets, Homeobox, forkhead TFs in fly
Wei_2010	PBM	20517297	16	Ets in mouse

Table S2 Coevolving residue pairs

Attached xls file (“Table S2.highly_coevolved_residues.xlsx”)

Table S3 Residue blocks in TF domains

TF Family	coevolving residue positions
HLH	[1] "13_14_15_16"<br>[2] "1_2_3_4_5_6_7_8_9_10_11_12"<br>[3] "19_20_21"<br>[4] "29_30_31_32_33_34_35_36_37_38_39_40_41_42_43"<br>[5] "45_46_47_48"
zf-C4	[1] "3_4_5_6_7_8_9_10_11_12_13_14_15_16_17_18_19_20_21_22_23"<br>[2] "30_31_32_33",<br>[3] "47_48_49_50_51_52_53_54"<br>[4] "54_55_56_57_58_59"<br>[5] "37_38_39"
bZIP_1	[1] "1_2_3_4_5_6_7"<br>[2] "10_11_12"<br>[3] "23_24_25_26_27_28_29_30"<br>[4] "34_35_36_37_38_39_40_41_42_43_44_45_46_47"<br>[5] "53_54_55_56_57_58"<br>[6] "58_59_60_61_62_63_64"<br>[7] "17_18_19"
Zn_clus	[1] "14_15_16_17_18_19" "1_2_3_4_5_6_7_8_9"<br>[3] "24_25_26_27_28_29" "31_32_33_34_35_36_37_38_39_40"
Ets	[1] "30_31_32_33_34_35_36_37"<br>[2] "1_2_3_4_5_6_7_8_9_10_11_12_13_14_15_16_17_18_19_20"<br>[3] "23_24_25_26_27"<br>[4] "71_72_73_74_75_76_77_78_79_80_81"<br>[5] "61_62_63"<br>[6] "54_55_56"<br>[7] "65_66_67"
HMG_box	[1] "17_18_19_20_21_22_23_24_25_26_27_28_29_30_31_32_33"<br>[2] "51_52_53_54_55_56_57_58_59_60_61_62"<br>[3] "63_64_65_66_67_68_69"<br>[4] "1_2_3_4_5_6_7_8_9_10_11_12_13_14_15"<br>[5] "45_46_47"

Table S4. Representative 3D structures of TF-DNA complex

TF Family	coevolving residue positions
HLH	4H10, 1NLW, 1AN2, 1HLO, 1NKP, 4ATI, 4ATK,1MDY, 2QL2, 1A0A, 1AM9, 2YPA, 2YPB, 1AN4
zf-C4	1R4I, 1R0N, 1R0O, 2HAN, 1HCQ, 4AA6, 4HN5, 4HN6, 1GLU, 1LAT, 1R4O, 1R4R, 3FYL, 3G6P, 3G6Q, 3G6R, 3G6T, 3G6U, 3G8U, 3G8X, 3G97, 3G99, 3G9I, 3G9J, 3G9M, 3G9O, 3G9P, 3CBB, 4IQR, 4TNT, 1A6Y, 1GA5, 1HLZ, 1CIT, 2A66, 3DZU, 3DZY, 3E00, 1DSZ, 1BY4, 4CN2, 4CN3, 4CN5, 4CN7, 4NQA, 1YNW, 2NLL, 3M9E, 1KB2, 1KB4, 1KB6
bZIP_1	1H8A, 1H89, 1H88, 1NWQ, 1JNM, 1GU5, 1GU4, 1GTW, 2E43, 2E42, 3A5T, 2WT7, 2WTY, 4AUW, 1HJC, 1GD2, 2DGC
Zn_clus	1D66, 3COQ, 1HWT, 1QP9, 2HAP, 2ER8, 2ERE, 2ERG, 1PYI, 1ZME
Ets	3JTG, 1DUX, 1BC7, 1BC8, 1HBX, 1K6O, 4IRI, 2NNY, 3MFK, 3RI4, 3WTS, 3WTT, 3WTU, 3WTV, 3WTW, 3WTX, 3WTY, 3WU1, 4L0Y, 4L0Z, 4L18, 4LG0, 1K78, 1K79, 1K7A, 1MDM, 4BQA, 4BNC, 4UUV, 4UNO, 4MHG, 3ZP5, 5E8I, 5JVT, 1AWC, 1YO5, 1PUE
HMG_box	1QRV, 3NM9, 3F27, 4Y60, 3U2B, 4EUW, 4S2Q, 3TMM, 3TQ6, 4NNU, 4NOD
Homeobox	3A01, 3LNQ, 9ANT, 4RDU, 1JGG, 1B8I, 2R5Y, 2R5Z, 4CYC, 4UUS, 4J19, 2HDD, 2HOS, 2HOT, 1DU0, 1HDD, 3HDD, 1IC8, 2H8R, 1PUF, 1B72, 1IG7, 1AKH, 1APL, 1MNM, 1YRN, 1K61, 1LE8, 4RBO, 3RKQ, 3CMY, 1AU7, 1CQT, 2XSD, 3L1P, 3D1N, 1FJL, 4S0H
